# Patients with atrial fibrillation and common exclusion criteria from clinical trials are at high risk of clinical events: the Murcia AF Project II (MAFP-II) cohort study

**DOI:** 10.1007/s11739-024-03701-9

**Published:** 2024-07-04

**Authors:** Eva Soler-Espejo, José Miguel Rivera-Caravaca, José Daniel Bru-Cánovas, María Asunción Esteve-Pastor, Gregory Yoke Hong Lip, Francisco Marín, Vanessa Roldán

**Affiliations:** 1 Department of Hematology, Hospital Clínico Universitario Virgen de La Arrixaca, University of Murcia, Instituto Murciano de Investigación Biosanitaria (IMIB-Arrixaca), Murcia, Spain; 2 Faculty of Nursing, University of Murcia, Murcia, Spain; 3 Liverpool Centre of Cardiovascular Science at University of Liverpool, Liverpool John Moores University and Liverpool Heart and Chest Hospital, Liverpool, UK; 4 Faculty of Medicine, University of Murcia, Murcia, Spain; 5 Department of Cardiology, Hospital Clínico Universitario Virgen de La Arrixaca, University of Murcia, Instituto Murciano de Investigación Biosanitaria (IMIB-Arrixaca), CIBERCV, Murcia, Spain; 6 Department of Clinical Medicine, Aalborg University, Aalborg, Denmark

**Keywords:** Atrial fibrillation, Exclusion criteria, Stroke, MACE, Mortality, Major bleeding

## Abstract

**Background:**

Some clinical characteristics and comorbidities in atrial fibrillation (AF) patients are exclusion criteria in randomized clinical trials (RCTs) investigating oral anticoagulants (OAC). However, these conditions are present also in everyday clinical practice patients. We compared the risk of adverse clinical outcomes between patients with and without RCT exclusion criteria.

**Methods:**

The Murcia AF Project II was an observational cohort study including AF outpatients starting vitamin K antagonists (VKAs) from July 2016 to June 2018. For the selection of the exclusion criteria, the four pivotal RCTs of direct-acting OAC (DOACs) were used as reference. During 2 years, all ischemic strokes/transient ischemic attacks, major adverse cardiovascular events (MACEs), major bleeds, and all-cause deaths were recorded.

**Results:**

1050 patients (51.5% female, median age 77 years) were included, of whom 368 (35%) met at least one exclusion criterion for RCTs. During follow-up, the incidence rate ratios for major bleeding, MACE and all-cause mortality were higher among patients with exclusion criteria (all *p* < 0.001). Patients fulfilling at least one exclusion criterion had increased risks of major bleeding (aHR 1.48; 95% CI 1.22–1.81; *p* < 0.001), MACE (aHR 1.51, 95% CI 1.10–2.09, *p* = 0.012), and mortality (aHR 3.22, 95% CI 2.32–4.48, *p* < 0.001), as well as a lower event-free survival (all log-rank *p* < 0.001).

**Conclusions:**

In this AF cohort taking VKAs, more than one-third had at least one RCT exclusion criteria, which translates into higher risk of major bleeding, MACE, and death. These observations should be considered when translating RCTs results to AF patients for a proper and a more patient-centered management.

**Supplementary Information:**

The online version contains supplementary material available at 10.1007/s11739-024-03701-9.

## Introduction

Atrial fibrillation (AF) is the most common arrhythmia, with an estimated prevalence of about 1–2% in the overall population and up to 15% in the elderly aged over 80 years of age [1]. Multiple risk factors are associated with an increased likelihood of AF development, such as hypertension, valvular heart disease, heart failure, ischemic heart disease and sleep apnea [2]. Hence, AF patients are commonly multi-morbid, with frailty, polypharmacy and clinical complexity, with implications for treatment and prognosis [3–5]. The current holistic or integrated care approach to AF management [6], which is associated with improved clinical outcomes [7], is therefore recommended in guidelines [8, 9].

Reducing the risk of risk of stroke and thromboembolism using appropriate oral anticoagulation (OAC) therapy is one the fundamental pillars of the management of AF. Several studies have shown that direct-acting oral anticoagulants (DOACs) avoid most of the drawbacks of vitamin K antagonists (VKAs), but there is still scarce evidence about their efficacy and safety in certain subpopulations. Indeed, the pivotal DOAC randomized clinical trials (RCTs) were characterized by strict inclusion and exclusion criteria [10–13].

Although the strongest scientific evidence is derived from RCTs, these studies are not free of limitations, such as a careful patient selection and exclusion criteria. The reality is that in daily clinical practice, patients are much more heterogeneous than in RCTs and present with comorbidities that would be considered as an exclusion criterion for RCT enrollment. Therefore, there is a need to validate the event rates seen in RCTs in observational studies to investigate how these clinical characteristics influence adverse events and verify if RCT results could be extrapolated to daily clinical practice. Herein, we aimed to investigate whether the clinical characteristics of AF patients who meet RCT exclusion criteria are different from those of patients who do not meet exclusion criteria, and second, to compare the risk of adverse events between these two groups.

## Methods

This was a prospective observational cohort study including 1,050 outpatients diagnosed recently with any type AF (either paroxysmal, persistent, long-term persistent or permanent) and naïve for OAC, starting VKA therapy at our anticoagulation clinic of a tertiary hospital (Murcia, Spain) from July 1, 2016, to June 30, 2018. Only adult patients were included. Patients with prosthetic heart valves, rheumatic mitral valves or other severe valvular disease were excluded, with no other exclusion criteria. A more detailed description of the methods and the Murcia AF Project II (MAFP-II) cohort has been previously published [14]. The present is post hoc analysis of an all-comers design study. Hence, no sample size calculations were performed.

At baseline, a complete medical history was obtained by collecting socio-demographic and anthropometric data, comorbidities, and concomitant therapies. In addition, stroke (CHA_2_DS_2_-VASc) and bleeding (HAS-BLED) risk scores were calculated.

The study protocol was approved by the Ethics Committee from the University Hospital Morales Meseguer (reference: EST: 20/16) and carried out in accordance with the ethical standards established in the 1964 Declaration of Helsinki its subsequent amendments. Informed consent was required to participation in this study.

### Definition of exclusion criteria for RCT

Patients were classified according to the presence, at study baseline, of exclusion criteria commonly established in the RE-LY, ARISTOTLE, ROCKET-AF, and ENGAGE AF-TIMI 48 trials [10–13]. Patients were classified as “meeting exclusion criteria” if they fulfilled at least one of the following:

i. Moderate–severe anemia (defined as a hemoglobin < 100 g/L).
ii. Severe renal failure (estimated creatinine clearance ≤ 30 mL/min) or serum creatinine > 2.5 mg/dL.
iii. Uncontrolled hypertension (systolic blood pressure > 180 mm Hg or diastolic blood pressure > 100 mm Hg).
iv. Concurrent treatment with aspirin and a thienopyridine (e.g., clopidogrel, ticlopidine).
v. Platelet count < 90,000/μL.
vi. Frailty: severe comorbid condition with life expectancy ≤ 1 year.
vii. Active alcohol abuse.
viii. Recent severe stroke (< 6 months).

### Follow-up and clinical outcomes

Follow-up was for 2 years. During this period, ischemic stroke/transient ischemic attack (TIA), major bleeding (according to the 2005 International Society on Thrombosis and Haemostasis [ISTH] criteria) [15], major adverse cardiovascular events (MACE, defined as the composite of ischemic stroke/TIA, myocardial infarction or cardiovascular death), and all-cause mortality were the *primary endpoints.* The investigators identified, confirmed, and recorded all clinical outcomes. Of note, no patient was lost to follow-up.

### Statistical analysis

Quantitative variables were expressed as mean ± standard deviation (SD) or median and interquartile range (IQR) as appropriate, while categorical variables were expressed as absolute frequencies and percentages. Pearson’s chi-square test was used to compare proportions, and differences between quantitative and categorical variables were assessed using the Mann–Whitney *U* test or Student *t* test as appropriate.

Incidence rates with their Poisson 95% confidence intervals (CI) for primary outcomes during the follow-up were calculated in patients with and without exclusion criteria. Incidence rates were then compared and reported as incidence rate ratios (IRRs), with “no exclusion criteria” as the reference category.

Adjusted Cox proportional hazard regression models were performed to determine the independent risk of the primary outcomes based on the sum of exclusion criteria, on the presence of at least one exclusion criteria, and on the presence of ≥ 2 exclusion criteria. Results were expressed as adjusted hazard ratio (aHR) with the corresponding 95% CI. Survival analyses were performed using Kaplan–Meier curves, testing the difference by the log-rank test.

A p value < 0.05 was accepted as statistically significant. Statistical analyses were performed with SPSS v. 25.0 (SPSS, Inc., Chicago, IL, USA) and MedCalc v. 16.4.3 (MedCalc Software bvba, Ostend, Belgium) for Windows.

## Results

**Fig. 1:**
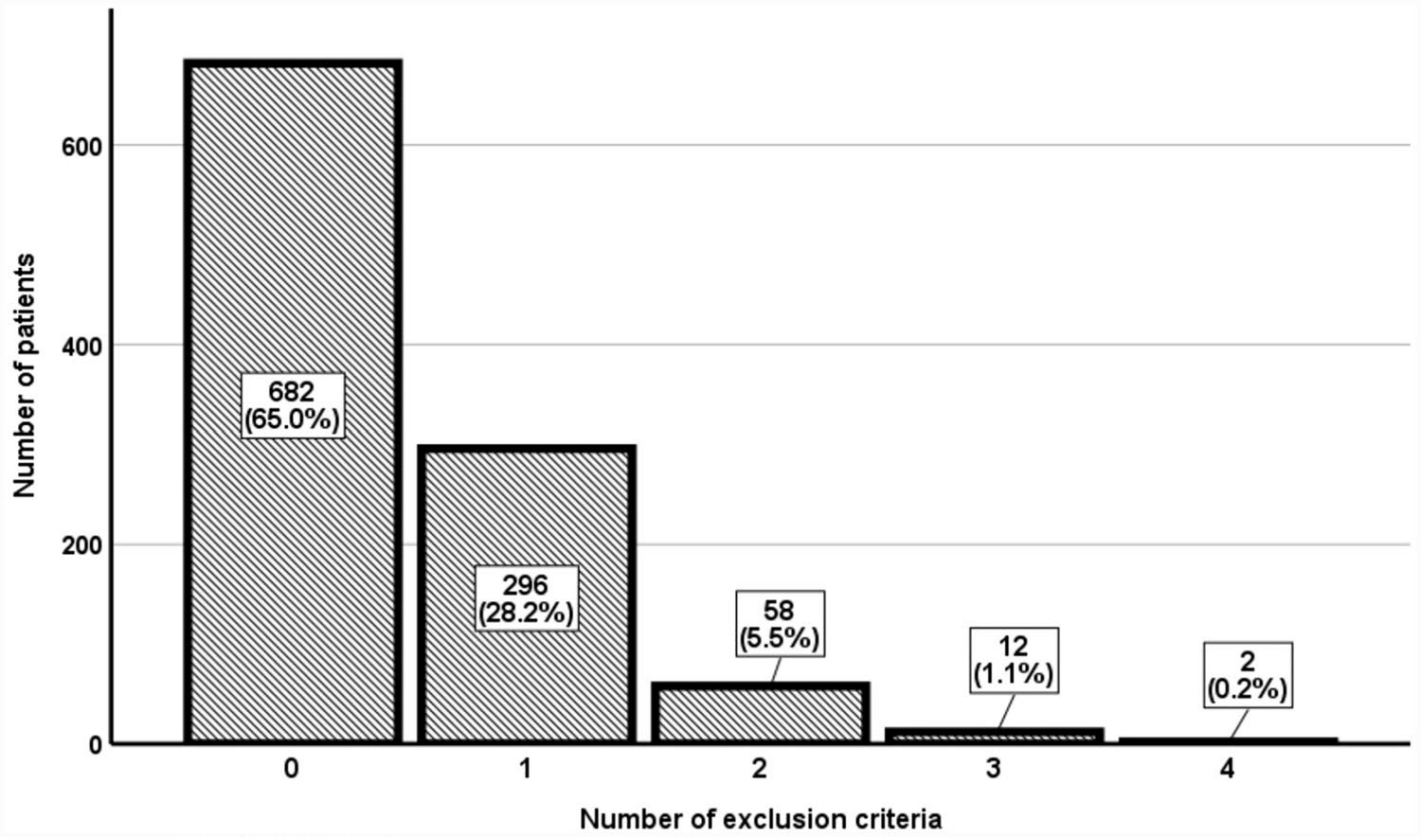
Distribution of patients based on the number of exclusion criteria

A total of 1050 AF patients (51.5% female, median age 77, IQR 69–83 years) were included, with a median CHA_2_DS_2_-VASc of 4 (IQR 3–5) and a median HAS-BLED of 2 (IQR 2–3). Of these, 682 (65%) had no RCT exclusion criteria, and 368 (35%) patients met at least one RCT exclusion criterion. The distribution of patients according to the number of exclusion criteria is detailed in Fig. 1 and the proportion of patients with different exclusion criteria is presented in the Supplementary Table 1.

Patients meeting at least one exclusion criteria were older and characterized by a greater presence of comorbidities as observed in Table 1. As a result, patients with at least one exclusion criteria had significantly higher median CHA_2_DS_2_-VASc (4 [IQR 3–6] *vs*. 4 [IQR 3–5]; *p* < 0.001) and HAS-BLED (3 [IQR 2–4] *vs*. 2 [IQR 2–3]; *p* < 0.001) scores. Additionally, the time in therapeutic range (TTR) was significantly lower in the group with at least one exclusion criteria (60.3% [IQR 47–74] *vs*. (65.7% [IQR 51–80]; *p* < 0.001).

**Table 1:** Baseline clinical characteristics

	Patients with no exclusion criteria (*N* = 682)	Patients with at least one exclusion criteria (*N* = 368)	*p* value
Demographics			
Age (years), median (IQR)	76 (69–82)	79 (71–85)	< 0.001
Female, *n* (%)	357 (52.3)	183 (49.7)	0.418
Comorbidities, *n* (%)			
Hypertension	560 (82.1)	319 (86.7)	0.056
Diabetes mellitus	224 (32.8)	171 (46.5)	< 0.001
Heart failure	136 (19.9)	127 (34.5)	< 0.001
History of ischemic stroke/TIA	82 (12.0)	81 (22.0)	< 0.001
Vascular disease^a^	131 (19.2)	100 (27.2)	0.003
Renal impairment	84 (12.3)	113 (30.7)	< 0.001
Hypercholesterolemia	389 (57.0)	224 (60.9)	0.224
COPD/OSA	140 (20.5)	91 (24.7)	0.117
History of relevant bleeding	100 (14.7)	74 (20.1)	0.024
Liver disease	33 (4.8)	36 (9.8)	0.002
Cancer	88 (12.9)	62 (16.8)	0.081
Smoking habit	84 (12.3)	75 (20.4)	0.001
Concomitant treatment, *n* (%)			
Antiarrhythmics	81 (11.9)	43 (11.7)	0.927
ACE inhibitors	165 (24.2)	94 (25.5)	0.628
ARBs	304 (44.6)	152 (41.3)	0.308
Calcium channel blockers	200 (29.3)	120 (32.6)	0.270
Beta-blockers	480 (70.4)	246 (66.8)	0.237
Diuretics	310 (45.5)	264 (71.7)	< 0.001
Antilipemic agents	354 (51.9)	204 (55.4)	0.274
Oral hypoglycemic agents	139 (20.4)	113 (30.7)	< 0.001
Insulin	36 (5.3)	55 (14.9)	< 0.001
Antiplatelet therapy	134 (19.6)	119 (32.3)	< 0.001
Aspirin alone	113 (16.5)	74 (20.1)	
Clopidogrel alone	21 (3.1)	22 (6.0)	
Dual antiplatelet therapy	0 (0.0)	23 (6.3)	
Time in therapeutic range (%), median (IQR)	65.7 (51–80)	60.3 (47–74)	< 0.001

*IQR* interquartile range, *TIA* transient ischemic attack, *COPD/OSA* chronic obstructive pulmonary disease/obstructive sleep apnea, *ACE inhibitors* angiotensin-converting-enzyme inhibitors, *ARBs* angiotensin II receptors blockers

^a^Vascular disease includes coronary artery disease and/or peripheral artery disease

### Primary adverse events and relationship to RCT exclusion criteria

During a median follow-up of 2 years, 67 (6.4%) patients suffered an ischemic stroke/TIA; 64 (6.1%) had a major bleeding event; 65 (15.7%) suffered from any of the MACE components; and 173 (16.5%) died. The IRRs for major bleeding, MACE, and all-cause mortality were significantly higher among patients with any exclusion criteria (all *p* < 0.001), but the IRR of ischemic stroke/TIA was non-significantly different. Detailed comparisons are shown in Table 2.

**Table 2:** Incidence rate and incidence rate ratio of the different outcomes among periods

	Patients with no exclusion criteria (*N* = 682)		Patients with at least one exclusion criteria (*N* = 368)		Incidence rate ratio (95% CI)	*p* value
	*N* (%)	Incidence rate (95% CI)	*N* (%)	Incidence rate (95% CI)		
Ischemic stroke/TIA	42 (6.2)	3.08 (2.22–4.16)	25 (6.8)	3.40 (2.20–5.01)	1.10 (0.64–1.85)	0.692
Major bleeding	24 (3.5)	1.76 (1.13–2.62)	40 (10.9)	5.43 (3.88–7.40)	3.09 (1.82–5.36)	< 0.001
MACE	83 (12.2)	6.09 (4.85–7.54)	82 (22.3)	11.14 (8.86–13.83)	1.83 (1.33–2.51)	< 0.001
Mortality	59 (8.7)	4.32 (3.29–5.58)	114 (31.0)	15.50 (12.78–18.61)	3.58 (2.59–4.99)	< 0.001

*CI* confidence interval, *MACE* major adverse cardiovascular events, *TIA* transient ischemic attack

Notwithstanding the low numbers and wide 95% CIs, there was no statistical difference for ischemic stroke/TIA in relation to RCT exclusion criteria either for the number of accumulated exclusion criterion met (HR 1.01, 95% CI 0.70–1.45, *p* = 0.940), or according to meeting at least one criterion (HR 1.24, 95% CI 0.75–2.03, *p* = 0.401) or ≥ 2 criteria (HR 0.503, 95% CI 0.12–2.06, *p* = 0.339).

Cox regression showed that patients had a significantly higher risk of major bleeding for each exclusion criterion, even after adjusting for HAS-BLED score (aHR 1.90, 95% CI 1.44–2.50, *p* < 0.001). The risk of major bleeding was significantly higher in patients meeting at least one exclusion criteria compared to those without exclusion criteria (aHR 1.48; 95% CI 1.22–1.81; *p* < 0.001), and in those fulfilling at least two exclusion criteria (aHR 2.94; 95% CI 1.52–5.70; *p* = 0.001).

For MACE, there was an increased risk per additional exclusion criterion in the model adjusted by CHA_2_DS_2_-VASc and HAS-BLED scores (aHR 1.29, 95% CI 1.05–1.58, *p* = 0.015). In turn, patients with at least one exclusion criterion had 50% increased risk of MACE (aHR 1.51, 95% CI 1.10–2.09, *p* = 0.012), whereas patients with ≥ 2 criteria exclusion criteria had a non-significantly increase in risk (aHR 1.35, 95% CI 0.81–2.25, *p* = 0.255).

The risk of death was significantly higher as the number of RCT exclusion criteria increased on Cox regression analysis adjusted by CHA_2_DS_2_-VASc and HAS-BLED (HR 1.86, 95% CI 1.58–2.19, *p* < 0.001). Patients with at least one exclusion criterion had a threefold higher risk of death (aHR 3.22, 95% CI 2.32–4.48, *p* < 0.001), and those with at least two exclusion criteria were also at increased risk (aHR 2.58, 95% CI 1.72–3.87, *p* < 0.001).

**Fig. 2:**
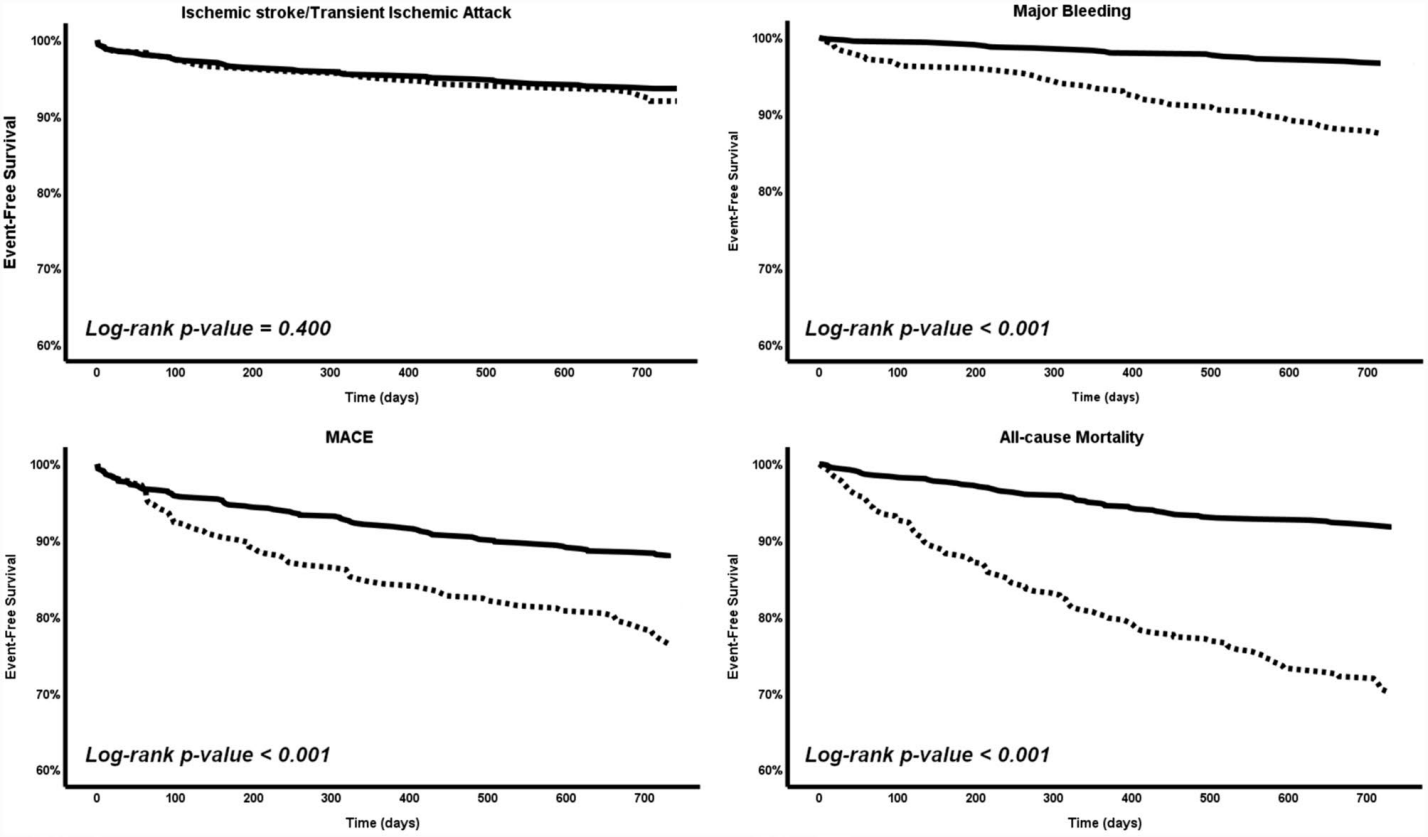
Kaplan–Meier survival curves for the primary outcomes. Solid black line = patients with no exclusion criteria. Dashed black line = patients with at least one exclusion criteria

Kaplan–Meier analyses showed that patients with exclusion criteria had lower event-free survival from major bleeding (log-rank *p* < 0.001), MACE (log-rank *p* < 0.001), and all-cause mortality (log-rank *p* < 0.001), but not from ischemic stroke/TIA (log-rank *p* = 0.400) (Fig. 2).

As there were significant differences in median TTR between both groups of patients, a sub-analysis adjusting also for TTR in the Cox regression analyses was performed (Supplementary Table 2). Overall, the results were similar to the main analysis and the risk of major bleeding, MACE and all-cause death was significantly increased in patients with at least one exclusion criterion compared to those without exclusion criteria, even accounting for the quality of VKA treatment (aHRs of 2.37, 1.43 and 2.50, respectively).

## Discussion

In this prospective cohort study, our principal findings are as follows: (i) one-third of AF patients met some common RCT exclusion criteria, and thus, these patients would be systematically excluded from RCT participation; (ii) patients who met exclusion criteria in RCTs had a higher risk profile, and greater comorbidities; and (iii) the presence of exclusion criteria was associated with an increased risk of major bleeding, MACE, and mortality, which was even higher *per* with each additional exclusion criteria.

Stroke prevention with OAC is an essential part of the management of AF [8, 9]. However, there remains a significant proportion of patients in whom the management of OAC is particularly challenging. This may be because many of the studies and clinical trials on OAC established strict inclusion and exclusion criteria, resulting in under-representation of such patients who are usually managed in everyday clinical practice with limited evidence-based support, but leading to uncertainties and lack of information when trying to extrapolate the results of RCTs to everyday clinical practice.

Therefore, although RCTs provide the highest level of scientific evidence, observational studies would supplement the evidence gap in this field. For this reason, there should be an increase in the number of pragmatic clinical trials where participants resemble the less selected populations that would benefit from the treatments and interventions under investigation. In pragmatic trials, inclusion and exclusion criteria should be reduced as much as possible, and both the number of reviews and the completeness of patient follow-up should be increased, given the trends toward more personalized medicine. This approach allows the results of pragmatic trials to closely resemble daily clinical practice [16].

Undoubtedly, the presence of cardiovascular risk factors adds to the clinical complexity and challenges of AF management. Conditions such as thrombocytopenia may indicate the presence of comorbidities associated with poor survival in AF [17], and acts as an independent risk factor for bleeding [18]. There are even additional exclusion criteria beyond those considered in this study. For instance, patients with liver dysfunction were excluded from all RCTs comparing DOACs to warfarin due to their higher risk of both thrombosis and bleeding compared to AF patients without liver disease, thereby creating a significant knowledge gap [19]. Indeed, liver disease and fibrosis have shown to increase the risk of worse clinical outcomes, particularly in VKA users [20–22]. Despite the high risk of adverse outcomes in these complex patients, anticoagulation is under-prescribed and discontinuations are common [3]. DOACs offer a better safety and effectiveness profile, but the scientific evidence from RCTs is limited as these patients are less likely to have been included in a clinical trial.

The insights gleaned from post-authorization studies provide several significant benefits for patients undergoing treatment with DOACs. These studies serve as valuable complements to earlier-phase clinical trials, providing additional data on the safety and efficacy of these medications in less restricted scenarios. For example, the ETNA-AF and XANTUS studies confirmed the safety and efficacy of edoxaban and rivaroxaban over warfarin in clinical practice settings. Notably, their findings are even more reassuring than those from their respective pivotal trials. The broader patient profiles observed in these studies suggest a wider applicability of DOACs compared to the more stringent inclusion criteria of phase III trials [23]. Moreover, the comorbidity profiles of patients in these studies closely resemble those in our own research.

We previously demonstrated that everyday clinical practice patients have inherent factors that make them different from patients included in an RCT, and are at a higher risk of adverse events when compared to RCT patients, even if differences are appropriately adjusted using statistical methods [24]. Similarly, a study showed that risk profiles are higher in AF patients included in observational studies compared to patients included in RCTs, and the higher rates of bleeding and mortality not attributable to thromboembolism in these patients suggest that there are additional differences in their characteristics that are important contributors to this difference in event rates [25]. Another recent study showed that in elderly patients with venous thromboembolism, those with ≥ 2 exclusion criteria had a twofold (sHR 2.16, 95% CI 1.38–3.39) increased risk of major bleeding compared to eligible patients, and the bleeding risk increases significantly with the number of exclusion criteria present [26]. On the contrary, Steinberg et al*.* found that patients treated with OACs and contraindication to anticoagulation had a lower risk of stroke, hospitalization, and death although there was an increased risk of intracranial hemorrhage. In addition, they showed that even in cases of blood dyscrasias and intracranial bleeding, the net clinical benefit was still positive, hence demonstrating that a high risk of bleeding should not be an absolute contraindication to OAC as many of the risk factors for bleeding are also risk factors for stroke [27].

Certainly, many comorbidities, including anemia, thrombocytopenia, renal failure, or uncontrolled hypertension do not represent an absolute contraindication to OAC. Therefore, future RCTs should reduce the number of exclusion criteria so that they can provide a more objective and less restrictive scenario, thus providing a more realistic picture and broadening the generalizability of study results. In parallel, it would be desirable to investigate which specific exclusion criteria are associated with a higher risk of events, in order to design specific studies in such populations.

### Limitations

There are some limitations in relation to this study that should be acknowledged. The main one lies in its observational nature, with a Caucasian-based population and single-center design. Another potential limitation is patient selection because only those patients who initiated VKA therapy were included as this was the only OAC authorized for OAC-naïve patients in Spain. Thus, we need to recognize that our results could not apply to a DOAC-treated population, where the risk of several outcomes could be attenuated by the greater effectiveness and safety of DOACs.

In addition, we acknowledge that the definitions of stroke exclusion criteria are not exactly the same between the four DOAC RCTs (RE-LY: strokes within the previous 14 days were excluded [6 months for severe stroke]; ARISTOTLE: strokes within the previous 7 days were excluded; ROCKET AF: strokes within the previous 14 days were excluded [3 months for severe stroke]; ENGAGE TIMI AF: strokes within the previous 30 days were excluded). Hence, we decided to use the 6 months timing for severe strokes.

Finally, although we included severe frailty condition as a criterion, we did not consider other subjective exclusion criteria such as “the investigator’s opinion that a subject would have an unacceptable risk from study participation, a suboptimal compliance, or any condition precluding successful study completion”. It is therefore likely that the real proportion of non-eligible patients for a RCT would be even higher.

## Conclusions

In this prospective cohort of AF patients taking VKA therapy, more than one-third of patients had at least one RCT exclusion criteria, which translates into an increased risk of major bleeding, MACE, and death. Our findings suggest that may be variations in the results of RCTs when applied to patients from everyday clinical practice and the risk profile could be difference. These issues should be considered when translating RCTs results to AF patients for a proper and a more patient-centered management.

## Supplementary Information

Below is the link to the electronic supplementary material.

Supplementary file1 (DOCX 26 KB)

## Data Availability

Derived data supporting the findings of this study are available from the corresponding author José Miguel Rivera-Caravaca on request.
