# Supplementary material for: Patients with atrial fibrillation and common exclusion criteria from clinical trials are at high risk of clinical events: the Murcia AF Project II (MAFP-II) cohort study
Source: Intern Emerg Med. 2024 Jul 4;19(7):1941–8. doi: 10.1007/s11739-024-03701-9 (PMC11467067; doi:10.1007/s11739-024-03701-9)
Supplement: Supplementary file 1 — Supplementary file1 (DOCX 26 KB) [file 11739_2024_3701_MOESM1_ESM.docx]

**Supplementary Material**

**Supplementary Table 1.** Proportion of patients with different exclusion criteria.

| **Exclusion criterion** | **N (%)** |
| --- | --- |
| Frailty: severe comorbid condition with life expectancy ≤1 year | 186 (50.5) |
| Active alcohol abuse | 72 (19.6) |
| Severe renal failure | 63 (17.1) |
| Moderate-severe anaemia | 60 (16.3) |
| Uncontrolled hypertension | 38 (10.3) |
| Concurrent treatment with aspirin and a thienopyridine | 21 (5.7) |
| Platelet count <90,000/μL | 15 (4.1) |
| Recent severe stroke (<6 months) | 11 (3.0) |

**Supplementary Table 2.** Cox regression analyses for the outcomes of interest adjusted by CHA_2_DS_2_-VASc, HAS-BLED and time in therapeutic range.

|  | **aHR** | **95% CI** | **p-value** |
| --- | --- | --- | --- |
| **Mayor Bleeding** | | | |
| For each exclusion criteria | 2.08 | 1.48-2.93 | <0.001 |
| Patients with at least one exclusion criteria | 2.37 | 1.28-4.42 | 0.006 |
| Patients with at least two exclusion criteria | 4.28 | 2.02-9.05 | <0.001 |
| **MACE** | | | |
| For each exclusion criteria | 1.19 | 0.94-1.50 | 0.146 |
| Patients with at least one exclusion criteria | 1.43 | 1.01-2.03 | 0.047 |
| Patients with at least two exclusion criteria | 0.93 | 0.48-1.79 | 0.822 |
| **All-cause mortality** | | | |
| For each exclusion criteria | 1.69 | 1.39-2.06 | <0.001 |
| Patients with at least one exclusion criteria | 2.50 | 1.74-3.60 | <0.001 |
| Patients with at least two exclusion criteria | 2.05 | 1.24-3.39 | 0.005 |
| aHR: adjusted hazard ratio; CI = confidence interval | | | |
